# Uncoupled phytoplankton-bacterioplankton relationship by multiple drivers interacting at different temporal scales in a high-mountain Mediterranean lake

**DOI:** 10.1038/s41598-019-57269-y

**Published:** 2020-01-15

**Authors:** Cristina Durán-Romero, Juan Manuel Medina-Sánchez, Presentación Carrillo

**Affiliations:** 1 Instituto del Agua, Universidad de Granada, E-18071 Granada, Spain; 2 Departamento de Ecología, Facultad de Ciencias, Universidad de Granada, E-18071 Granada, Spain

**Keywords:** Climate-change ecology, Food webs, Freshwater ecology, Microbial ecology, Limnology

## Abstract

Global-change stressors act under different timing, implying complexity and uncertainty in the study of interactive effects of multiple factors on planktonic communities. We manipulated three types of stressors acting in different time frames in an *in situ* experiment: ultraviolet radiation (UVR); phosphorus (P) concentration; temperature (T) in an oligotrophic Mediterranean high-mountain lake. The aim was to examine how the sensitivity of phytoplankton and bacterioplankton to UVR and their trophic relationship change under nutrient acclimation and abrupt temperature shifts. Phytoplankton and bacteria showed a common pattern of metabolic response to UVR × P addition interaction, with an increase in their production rates, although evidencing an inhibitory UVR effect on primary production (PP) but stimulatory on bacterial production (HBP). An abrupt T shift in plankton acclimated to UVR and P addition decreased the values of PP, evidencing an inhibitory UVR effect, whereas warming increased HBP and eliminated the UVR effect. The weakening of commensalistic and predatory relationship between phyto- and bacterioplankton under all experimental conditions denotes the negative effects of present and future global-change conditions on planktonic food webs towards impairing C flux within the microbial loop.

## Introduction

Global change is the result of multiple anthropic stressors that drive an accumulative impact on biodiversity and functioning of ecosystems^1^. Therefore, predictions of its effects on any ecosystem require assessments of the interactions among the abiotic stressors acting at regional scales (e.g. eutrophication, drought, atmospheric dust inputs, increased ultraviolet radiation [UVR]) as well as global ones (e.g. ozone depletion, global warming). The duration of stressors should be taken into account, as these can be chronic (e.g. UVR), pulsed [e.g. nutrient-pulses (pulse = combination of low frequency, large magnitude, and short duration, *sensu* Yang *et al*.^2^)] or abrupt (e.g. heat waves). Changes in these multiple factors may potentially trigger complex interactive effects among them; therefore, it is critical to determine their combined impact, which might be stronger (synergistic effect) or weaker (antagonistic effect) than the sum of their individual effects^3,4^. Besides the net effect on organisms, the magnitude and direction of the interaction of multiple factors might help in comparing organisms’ responses and in more fully understanding them^5,6^. However, for a complete and more realistic vision of the effects of multiple abiotic stressors on ecosystem, it is indispensable to consider the effects on relationships among species or trophic levels and changes in these interactions^7,8^.

One of the most sensitive regions in the world to the effects of climate change is the Mediterranean Basin, where the influence of multiple stressors tends to be accentuated^9,10^. Among Mediterranean ecosystems, high-mountain lakes are particularly vulnerable to global-change effects^11,12^ and therefore stand as good sentinels of environmental change at the local and global scales^13^. Among others, increased UVR fluxes in the Northern Hemisphere^14^ represent a worldwide chronic stressor, while a higher frequency of abrupt extreme weather events is expected to alter precipitation regimes and expand desertification in the Mediterranean region^15,16^. Consequently, pulsed nutrient loads of phosphorus (P) and nitrogen (N) may augment, altering the functioning of the pelagic ecosystems in oligotrophic lakes and marine environments^17^. The Mediterranean area is also expected to undergo increases in maximum, minimum, and mean air temperatures^18^ as well as in the frequency of extreme events, such as heat waves^19^. Despite that the temperature effects have been largely interpreted in terms of changes in mean values, some studies have explicitly acknowledged that changes in the incidence of extreme events and in their temporal variability can have profound effects on ecosystems^20,21^. In addition, the effects of inter-annual and decadal fluctuations in temperature, which can trigger local changes by several degrees Celsius (i.e. much larger than the expected mean change at the global scale^10^), remain almost unknown^22,23^.

Natural plankton communities are ideal experimental models for studying the consequences of global change at the ecosystem level, because these unicellular organisms are relatively easy to manipulate and have short generation times^16^. Particularly important are the responses of phyto- and bacterioplankton since, due to their position at the base of the food web, they are the main factors responsible for exerting an ecosystem-scale influence on fundamental processes in aquatic system^24,25^. Phyto- and bacterioplankton are naturally exposed to high UVR levels in the water column, and therefore subjected to their harmful direct effects^26^. This exposure is intensified in oligotrophic high-mountain lakes, due to their high-altitude location^27^ (i.e. high UVR-fluxes) and usually having waters highly transparent to UVR due to lower concentrations of dissolved organic matter (DOM)^28^. However, the net effect of UVR can become non-negative as stimulation of heterotrophic bacterial production (HBP) and photosynthetic activity by UVR have been reported in several studies^29,30^. These contrasting results might be due to the UVR interaction with other environmental factors^28,31^. Thus, greater nutrient availability can either reduce photoinhibition on phyto- and bacterioplankton^32,33^ or unmask UVR damage on phytoplankton or bacterioplankton^34^. Likewise, the interaction of UVR with rising temperature could have a positive influence by reducing photoinhibition on the bacterial and phytoplanktonic community^35,36^.

Few experiments have focused on the interactive effects of UVR, nutrients, and temperature on phytoplankton and/or bacterioplankton^37–39^, and only one report has described the effects of these three factors on phytoplankton-bacterioplankton relationship in Mediterranean high-mountain lakes^39^. Recently, in Sierra Nevada high-mountain lakes (southern Spain) has been found a shift in phytoplankton-bacterioplankton relationship from a dual commensalistic-bacterivory control exerted by phytoplankton^40^ to a predominant commensalism under conditions of higher temperature and nutrient inputs after a decade of global change^22^. Moreover, the strength of phytoplankton-bacterioplankton commensalistic relationship is quickly modulated by the interactive effect among UVR, nutrient inputs and warming by mismatching phytoplankton-bacteria coupling^39^.

The aim of the present study was to examine how the sensitivity of phytoplankton and bacterioplankton to UVR under nutrient pulses may be altered by extreme temperature shifts in a model high-mountain lake in the Mediterranean region. Based on reports in the literature on Mediterranean high-mountain lakes and current environmental change trends^10^, we hypothesised that the enhanced development of phytoplankton and bacteria by pulsed nutrient under UVR will be impaired if they undergo abrupt temperature variations related to the extreme cold or warming events characteristic of Mediterranean region^41^. We also hypothesised that, with abrupt variations of temperature under the joint action of UVR and nutrients inputs, a shift will occur in the phytoplankton-bacterioplankton relationship towards a decoupled commensalism leading to a change in microbial food web functioning.

These hypotheses were tested in two steps mimicking the temporal scale in which the different stressors act. Firstly, we determined the response of primary production (PP), HBP and algal and bacterial abundance to P addition under UVR exposure in mid-term incubation (1 wk), using P concentrations that simulated current inorganic nutrient pulses from allochthonous inputs^42,43^. Secondly, we evaluated the influence of temperature shift (T shift) on the responses of the communities previously acclimated to UVR and nutrients over the short term (12 h). We quantified the magnitude and nature (synergistic or antagonistic) of the interactive effects among UVR, nutrients, and temperature on PP, HBP, and cell abundance.

## Results

### Initial conditions

**Figure 1:**
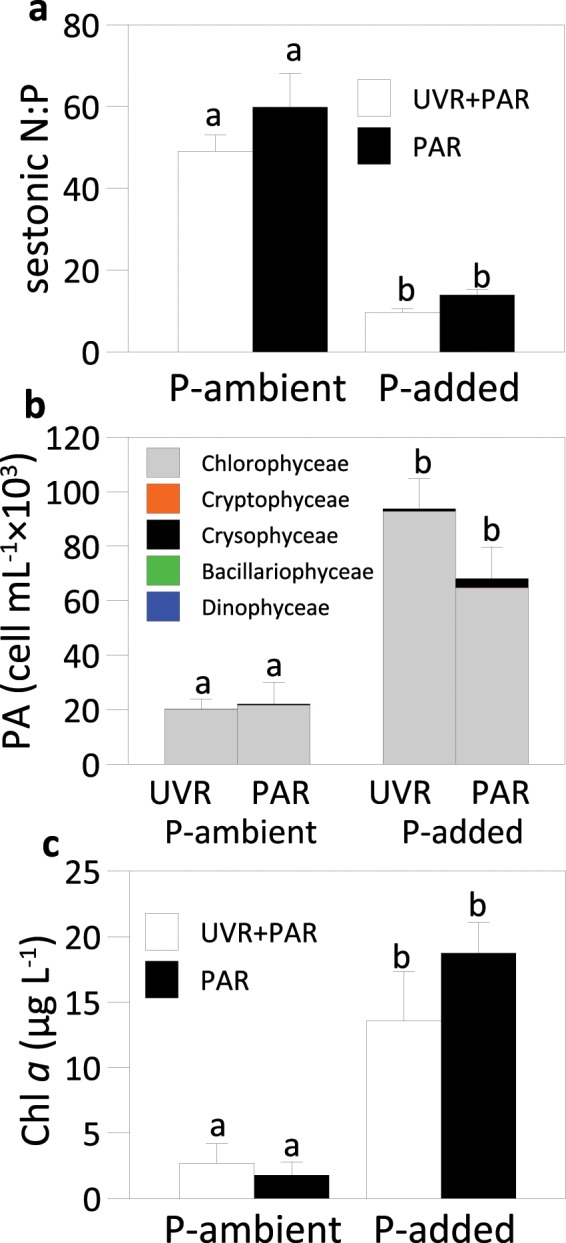
Sestonic N:P ratio (on a molar basis), phytoplankton abundance (PA) and chlorophyll *a* (Chl *a*) concentration under full sunlight (UVR + PAR) and photosynthetically active radiation (PAR) and under ambient phosphorus (P) concentration and P-added conditions. Bars represent the mean values and error bars represent the standard deviation (SD) (n = 3).

The water column showed low diffuse attenuation coefficients for downward irradiance (*k*_d_) for UV-B_305_ (0.33) and UV-A_380_ (0.17) with a near-surface UV-B_305_ irradiance of 0.035 W m^−2^ nm^−1^ (Fig. 1S). The surface temperature was 17.2 °C while the vertical profile was homogeneous (the temperature difference between surface and bottom was about 0.9 °C). La Caldera lake showed high dissolved inorganic nitrogen: total phosphorus ratio (DIN:TP) and sestonic N:P, both with values greater than the Redfielf ratio (Table 1S), suggesting a P limitation in the high-mountain lake. Bacterioplankton abundance (BA) was 5.76 ± 2.13 cell mL^−1^ × 10^5^ with a heterotrophic bacterial production of 0.04 ± 0.02 µg C L^−1^ h^−1^, whereas phytoplankton abundance (PA) was 4.88 ± 1.61 cell mL^−1^ × 10^3^ with a primary production of 2.02 ± 0.430 µg C L^−1^ h^−1^ (Table 1S). Non-flagellate phytoplankton was the dominant group, represented primarily by Chlorophyceae (87%, mainly *Monoraphidium* sp.)

### Interactive effects of UVR and P addition on phytoplankton and bacterioplankton and their modulation by T shift

After one week, the sestonic N:P ratio was higher than 40 in the treatments under P-ambient, i.e. similar to the initial N:P ratio. Meanwhile, the P addition decreased the sestonic N:P ratio (<20) in both radiation treatments (Fig. 1a) and, therefore, there was no significant interactive effect between radiation and nutrients on the N:P ratio (Table 2S). Under P-ambient and T_=_ conditions, UVR did not affect PA or chlorophyll *a* concentration (Chl *a*), which were stimulated by P addition but without reaching differences due to the radiation (Fig. 1b,c). Hence, radiation × nutrients interaction did not affect PA or Chl *a* (Table 2S). The phytoplankton composition barely changed with the treatments over the experiment (Fig. 1c).

**Figure 2:**
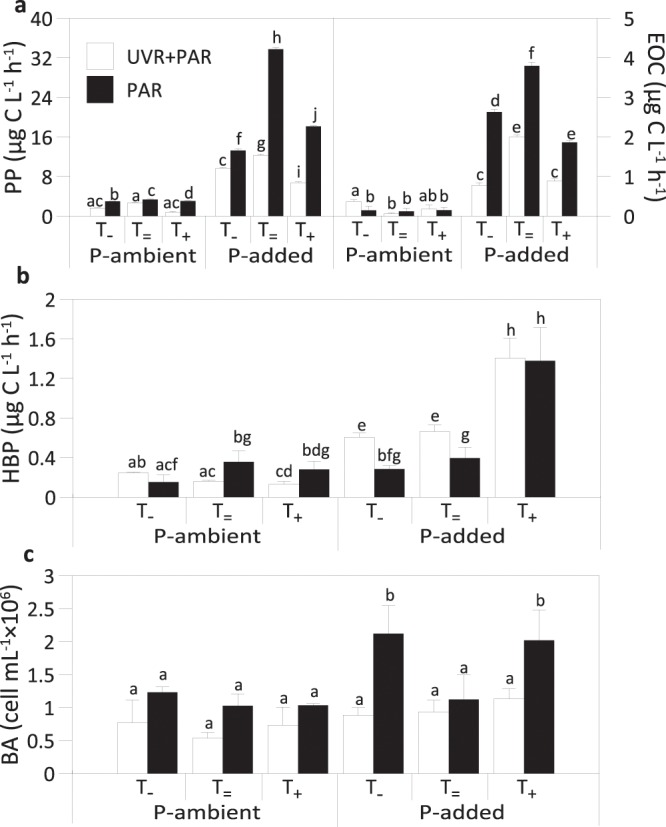
Primary production (PP) and algal-excreted organic carbon rates (EOC), heterotrophic bacterial production (HBP), and bacterial abundance (BA) under full sunlight (UVR + PAR) or photosynthetically active radiation (PAR), under ambient phosphorus (P) concentration or P-added conditions and under the three temperature treatments (T_−_: 5 °C below ambient temperature; T_=_: ambient temperature; T_+_: 5 °C above ambient temperature). Bars represent the mean values and error bars represent the standard deviation (SD) (n = 3). Significant differences among treatments are denoted by different lower-case letters.

Regarding functional variables, under P-ambient and T_=_ conditions, PP was significantly lower under the UVR + PAR than under the PAR treatment (Fig. 2a), resulting in an UVR inhibitory effect (Table 1). However, no differences were found between radiation treatments for excreted organic carbon (EOC) under these conditions (Fig. 2a). P addition raised the PP and EOC values up to 10-fold in the PAR treatment under T_=_ in comparison with the P-ambient treatment (Fig. 2a), which intensified the UVR inhibitory effect on PP but unmasked this effect on EOC (Table 1). Radiation, nutrients, and temperature exerted a significant interactive effect on PP and EOC rates (Table 3S). Thus, the T shift (warming as well as cooling) lowered the PP and EOC rates further in comparison with the ambient-temperature treatment (i.e. UVR + PAR × P-added × T_=_ treatment; Fig. 2a), although only the cooling weakened the UVR inhibitory effect generated by P addition on PP, but not on EOC rates (Table 1). The interactive effect among UVR + PAR, P addition, and T shift resulted in an antagonistic positive effect (following Piggot *et al*.^6^) for PP and EOC with a strength value of 70–80% (Table 2).

**Table 1:** Effect size of UVR (as percentage) on phytoplanktonic and bacterial variables under the treatments indicated. Different superscript letters indicate significant differences based on LSD *post hoc* test among the different treatments for phytoplanktonic and bacterial variables. PP: primary production; EOC: excreted organic carbon; HBP: heterotrophic bacterial production; BA: bacterial abundance. ns: not significant, indicates that differences between UVR and equivalent PAR treatment for each nutrient or nutrient × temperature treatment were not found (*p* > 0.05).

Treatment	Variable			
	PP	EOC	HBP	BA
UVR	−20^a^	ns	−55^a^	ns
UVR × T_+_	−75^b^	ns	ns	ns
UVR × T_−_	−44^c^	135^a^	ns	ns
UVR × P-added	−64^d^	−47^b^	68^b^	ns
UVR × P-added × T_+_	−63^d^	−52^b^	ns	−30^a^
UVR × P-added × T_−_	−27^e^	−70^b^	113^b^	−57^a^

**Table 2:** Types of interactive effects among ultraviolet radiation (UVR), phosphorus addition (P) and temperature (T)-shifts calculated from the magnitude and direction of the additive effect and interactive effect (in absolute terms) on algal and bacterial variables. ‘Control’ corresponds to response variable value in the PAR × P-ambient × T_=_ treatment; ‘UVR’ corresponds to the value in the UVR + PAR × P-ambient × T_=_ treatment; and ‘T’ to the value in PAR × P-ambient × T_+_ (in the case of warming) or PAR × P-ambient × T_−_ (in the case of cooling) treatments (See Fig. 2S). ‘Non-additive’ corresponds to the response-variable value in the UVR + PAR × P-added × T_+_ or UVR + PAR × P-added × T_−_ treatments for warming or cooling conditions respectively; and ‘Additive effect’ is the sum of control value plus the single UVR, P-addition and temperature shift effects. The strength of interactive effect represents the difference between the non-additive and additive effects divided by additive effect (as percentage). A: antagonistic interaction; S: synergistic interaction. The sign of the types of interactive effects and calculations are based on Piggot *et al*. (2015). PP: primary production; EOC: excreted organic carbon; HBP: heterotrophic bacterial production; BA: bacterial abundance. ^#^Variable response value × 10^6^. ns: no significant interactive effect among the three factors studied.

	Warming				Cooling			
	PP	EOC	HBP	BA	PP	EOC	HBP	BA
Control	3.37	0.12	0.36	1.02^#^	3.37	0.12	0.36	1.02^#^
UVR	2.68	0.06	0.16	0.53^#^	2.68	0.06	0.16	0.53^#^
P	33.7	3.79	0.40	1.12^#^	33.7	3.79	0.40	1.12^#^
T	3.06	0.14	0.28	1.03^#^	2.98	0.14	0.15	1.23^#^
Additive effect	34.7	3.76	0.13	1.62^#^	34.8	3.86	0.00	1.82^#^
Non-additive effect	6.76	0.88	1.41	1.13^#^	9.66	0.77	0.61	0.88^#^
**Interactive effect**	**+A**	**+A**	ns	**+S**	**+A**	**+A**	ns	**−A**
**Strengthen**	**−79.33**	**−76.53**		**76.56**	**−70.39**	**−79.47**		**4.76**

HBP was significantly lower under the UVR + PAR than under the PAR treatment at P-ambient and T_=_ conditions, resulting in an UVR inhibitory effect (Table 1); however, this effect was not significant on BA (Fig. 2b,c). P addition raised the HBP value up to 4-fold in the UVR treatment under T_=_ in comparison with the P-ambient treatment (Fig. 2b), resulting in a notable significant stimulatory UVR effect on HBP (Table 1). At the sub-plot level, significant interactive effect among radiation, nutrients, and temperature was found on BA but not on HBP (Table 3S). However, warming (T_+_ treatment) increased the HBP values under both radiation treatments in P-added samples (Fig. 2b), eliminating the UVR stimulatory effect found under T_=_ conditions (Table 1). Regarding to BA, after P addition, both cooling (T_−_ treatment) and warming (T_+_ treatment) increased abundance under PAR conditions (Fig. 2c), resulting in an inhibitory UVR effect (Table 1). The interactive effect among UVR + PAR, P addition and warming had a synergistically positive effect on BA with a strength value of 76%, whereas cooling had an antagonistic negative effect with a strength of 5% (Table 2).

**Figure 3:**
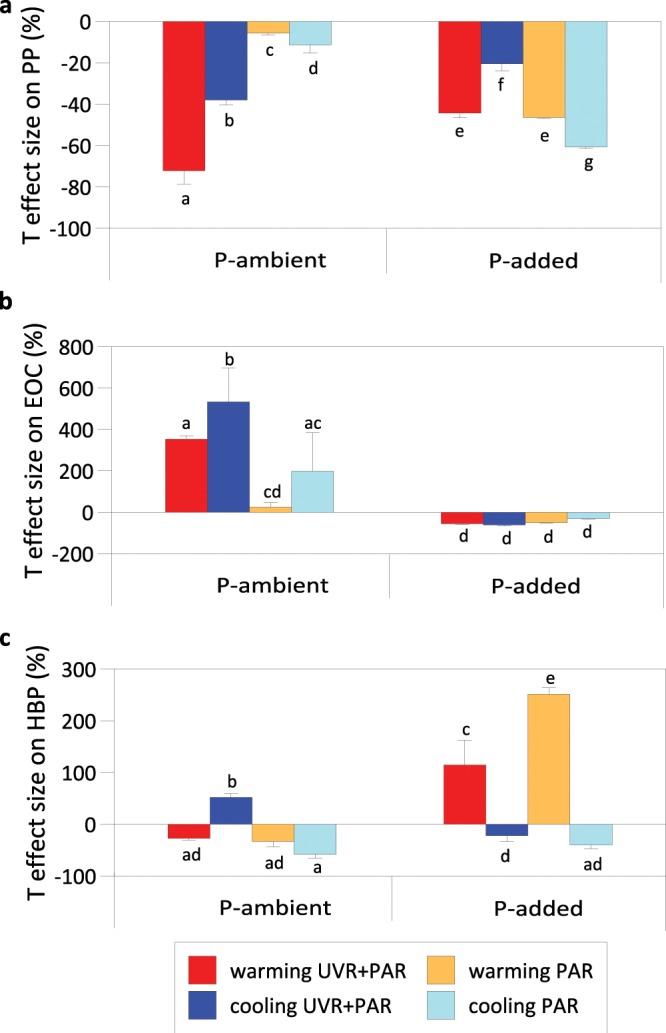
Temperature (T) effect size expressed as percentage on primary production (PP), algal-excreted organic carbon (EOC) rates and heterotrophic bacterial production (HBP) of plankton acclimated to full sunlight (UVR + PAR) or photosynthetically active radiation (PAR) and ambient phosphorus (P) concentration or P-added conditions. Warming: 5 °C above ambient temperature. Cooling: 5 °C below ambient temperature. Bars represent the mean values and error bars represent the standard deviation (SD) (n = 3). Significant differences among treatments are denoted by different lower-case letters.

The effect size of T shift on plankton communities acclimated to the radiation and nutrient treatments is summarized in Fig. 3. The T-shift exerted an inhibitory effect on PP under all treatments, although warming had a stronger effect than did cooling on (UVR + PAR)-acclimated phytoplankton whereas the opposite was found for (PAR)-acclimated phytoplankton (Fig. 3a). For (UVR + PAR)-acclimated phytoplankton, the inhibitory T effect on PP was weaker in P-added treatment in comparison with P-ambient conditions whereas the opposite occurred for PAR-adapted samples (Fig. 3a). In contrast to PP, warming had a stimulatory effect on EOC under P-ambient conditions, with a higher effect size on (UVR + PAR)- than on (PAR)-acclimated phytoplankton (Fig. 3b). On the other hand, phytoplankton adapted to P addition was inhibited by the T shift regardless of the radiation treatment (Fig. 3b). For bacterioplankton, cooling stimulated HBP on (UVR + PAR)-acclimated bacterioplankton under P-ambient conditions, and warming on bacterioplankton acclimated to P addition (Fig. 3c).

### Interactive effects of UVR and P addition on phytoplankton-bacterioplankton relationship and their modulation by T shift

**Figure 4:**
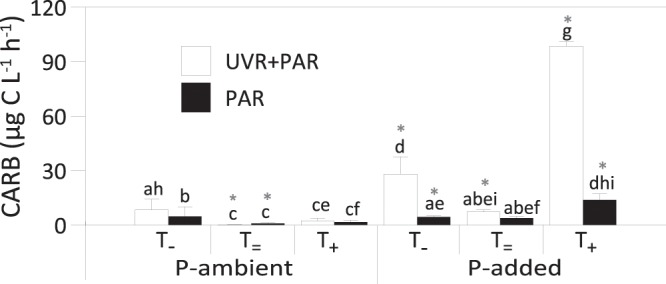
Photosynthetic carbon required by bacteria (CARB) under full sunlight (UVR + PAR) or photosynthetically active radiation (PAR), under ambient phosphorus (P) concentration or P-added conditions and under the three temperature treatments (T_−_: 5 °C below ambient temperature; T_=_: ambient temperature; T_+_: 5 °C above ambient temperature). Bars represent the mean values and error bars represent the standard deviation (SD) (n = 3). Significant differences among treatments are denoted by different lower-case letters. Asterisks denote significant differences between CARB values and algal-excreted organic carbon rates (EOC; see values in Fig. 2) under each experimental treatment from t-test analysis.

Regarding to phytoplankton-bacterioplankton relationship, no lineal relationship was found between HBP or BA and EOC (EOC: n = 12, r = 0.25, p = 0.43; BA: n = 12, r = 0.53, p = 0.07). Under non-manipulated nutrient and temperature conditions (P-ambient and T_=_), photosynthetic carbon required by bacteria (CARB) showed values of around 1 (PAR treatment) or lower than 1 (UVR + PAR treatment), CARB values being significantly higher than EOC values in these treatments (Fig. 4; t-test, PAR = p < 0.01, UVR + PAR = p < 0.05). Under T_=_ conditions, P addition increased CARB values in comparison with P-ambient conditions, these values being significantly higher than EOC under UVR + PAR treatment (Fig. 4; t-test, p < 0.05). Under P-added conditions, the T shift significantly increased CARB values in samples subjected to UVR + PAR treatment, these values being significantly higher than those of EOC for the same treatments (Fig. 4; t-test, p < 0.05 for all radiation × temperature treatments).

## Discussion

This study was designed to assess the vulnerability of phytoplankton and bacterioplankton communities from a Mediterranean high-mountain lake to the interactive effects of global-change stressors acting at different timing. This approach is in line with the current need for studies incorporating the temporal extent and action scales of environmental factors into research on multiple stressors, for a realistic evaluation of the complex effect of global change^21^.

In support of our first hypothesis, a net positive response of PP and HBP (i.e. higher production) to the joint action of UVR and P addition was found under ambient temperature. The net increase in PP due to P addition was reflected in greater phytoplankton abundance (higher growth) and a higher Chl *a* concentration. The stimulation of phytoplankton production and abundance by P addition is consistent with the unbalanced sestonic N:P ratio (≥40) under P-ambient, which reflects severe phytoplaktonic P limitation, in agreement with previous reports^44^. Notably, BA was not stimulated by P addition, suggesting a competitive advantage of phytoplankton over bacteria as reported in observational^40^ and experimental approaches in this lake^44,45^. The low abundance of mixotrophs and absence of ciliates in the lake during this experiment led us to disregard bacterivory as a major control mechanism of bacterial abundance, although we cannot rule out a constraint on BA development imposed by viral lysis^46,47^. Despite the net increase in PP under the joint action of UVR and P addition, P accentuated the negative UVR effect on PP and resulted in an increase in EOC. This result agrees with previous reports of the unmasking or negative effect of UVR after nutrient enrichment on PP^34,44,48,49^. The mechanism involved in the unmasking effect of UVR after P enrichment may be the photodamage caused by excessive electron flux with the activation of photosynthetic electron transport, given the absence of an efficient C-release mechanism (by eliminating the phosphoglycolate) to dissipate the reducing power of photosynthetic electron transport (see Carrillo *et al*.^49^). Nevertheless, higher UVR damage to cells by UVR exposure might also occur during the DNA synthesis (stimulated growth) under nutrient-replete conditions^50^. By contrast, the increased positive effect of UVR on HBP after the P pulse indicates a higher UVR tolerance of bacterioplankton than phytoplankton. Bacterial photorepair under UVR and P addition is the most plausible explanation of the stimulatory UVR effect on HBP detected, in accord with previous findings in La Caldera^29,33^. In addition, a balanced nutritional status after the P addition (N:P ratio ~ 20; Fig. 1a) could strengthen the light-independent mechanisms for repairing cell damage (e.g. nucleotide excision repair), which are ATP-dependent^51^. Furthermore, the increased P availability may prompt a more efficient utilization of dissolved organic carbon (DOC) by bacterioplankton, either from photolysis^52^, from algal EOC or viral lysis^47^. Although our experimental approach was not designed to evaluate dissolved organic-matter utilization by bacteria, the lack of a significant relationship between EOC and HBP in this experiment suggests that bacterial community under UVR and P addition was not primarily controlled by the availability of fresh-released photosynthetic carbon, but rather by other biotic controls, such as top-down control exerted mainly by lithic viral cycles^46,53^ that could break the bacterial dependence on EOC, usually evidenced in La Caldera^39,54^. In this line, previous findings also evidenced how the influence of other biotic components could also break phytoplankton-bacterioplankton coupling (bacterial dependence on phytoplanktonic carbon), such as the control on bacteria through P recycled by high zooplankton abundance^55^.

As mentioned above, extreme events can influence ecological responses more than the mean level of a given factor^21,56^, which justifies the use of extreme stressor levels in studies of potential climate-change impact. In this line, our findings show that abrupt T shift altered the net response of phytoplankton and bacterioplankton communities acclimated to the joint action of UVR and P addition. Thus, T shift (warming or cooling) reduced the value of PP found under the simultaneous effect of those factors whereas HBP was stimulated only under warming. Furthermore, cooling reduced the UVR inhibitory effect on PP in comparison with T_=_ conditions under P addition whereas warming eliminated the UVR stimulatory effect on HBP. These results contrast with previous findings in La Caldera lake, where simultaneous short-term action of warming and nutrients resulted in an increase in PP and lack of effect on HBP together with a weaker inhibitory UVR effect on PP but stronger on HBP^39^. These contrasting responses reflect the importance of considering the timing of stressors and the involvement of the nutritional status of plankton in order to cope with another environmental factor. This is evident in the present experiment, which used an acclimation period to nutrient input and an improvement of sestonic N:P before exposition to abrupt T shift. The harmful effect of T shift on PP may be attributable to a narrow thermal tolerance of the low diverse phytoplankton community in the high-mountain lake. Due to the phytoplankton generation time and the duration of the T shift event in our experiment, we noted no changes in phytoplankton community composition towards more temperature-tolerant species, as found in longer microcosm experiments^56,57^. Although we have no data on bacteria diversity, changes in bacterial (shorter generation time) composition, with increased biodiversity under warming and nutrient addition, might have been possible^58^, enabling the proliferation of rare bacterial phyla more tolerant to higher temperatures. Changes in bacterioplankton community composition frequently are mediated by phytoplankton response to environmental changes through EOC availability^59^. Thus, due to the lack of relationship between EOC and HBP, and the absence of significant changes in phytoplankton community composition, we might disregard remarkable indirect effects of the studied stressors on bacterial composition.

Comparing the interactive effects among UVR, P-addition and T shift on phyto- and bacterioplankton, we found that bacterioplankton would benefit more than phytoplankton under a scenario of global change. Thus, the positive strongly antagonistic nature of these factors on PP, measured by the procedure of Piggot *et al*.^6^, despite raising the value of production, intensified the harmful effect of UVR. Contrarily, for bacterioplankton, the non-interactive (additive) effect of UVR, P addition, and T shift on HBP, together with greater production, resulted in either a stimulatory or null effect of UVR.

With respect to the phytoplankton-bacterioplankton relationship, although previously a dual commensalistic-bacterivory control exerted by phytoplankton on bacterioplankton has been reported in La Caldera lake, a weakening of bacterivory in favour of commensalistic relationship has been evidenced in an observational study in Sierra Nevada lakes during the last decade ascribed to higher temperatures and nutrient inputs from dust aerosol depositions^22^. As mentioned above, we found a decoupled commensalistic relationship, with EOC values lower than bacterioplankton requirements in most treatments, signifying that organic C sources other than algal excretion should be used by bacterioplankton (see above). In our experiment, the low abundance of bacterivorous phytoplankton indicates the depression of the mixotrophic metabolism under present environmental conditions (current global change conditions) in comparison with historical reports for this lake (see Medina-Sánchez *et al*.^40^, Carrillo *et al*.^60^), but also under future expected conditions for the Mediterranean region (higher frequency of nutrient inputs and extreme temperature events). Although mixotrophy has been evidenced as an major ecological strategy in clear oligotrophic high-mountain lakes^61^, warming as well as more intense UV radiation (linked to lake stratification) and nutrient inputs favour the predominance of strict autotrophs over mixotrophs in phytoplankton community from these ecosystems^22,60^. The weakening of both commensalistic and predatory relationships means a reduction in C flux between phyto- and bacterioplankton and subsequently a weakening of the microbial loop.

Mediterranean high-mountain lakes appear to be particularly vulnerable to the combined action of global-change stressors. The rapid and sensitive response of microorganisms in high-mountain lakes make them useful as a warning system of environmental changes on local and global scales, supporting their role as sentinels of global change^13^. Thus, in La Caldera lake, bacterioplankton and phytoplankton showed a rapid response to interactive effect of global-change stressors acting under different timing. However, we found that bacterioplankton would benefit more than phytoplankton from a future expected scenario of global change, implying changes in the balance of heterotrophic vs. autotrophic metabolism towards higher heterotrophy and a reduction of the dependence of bacterioplankton on C from algal excretion.

## Methods

### Study site

The study was performed in La Caldera, a high-mountain lake above the tree line at an elevation of 3050 m.a.s.l. in Sierra Nevada National Park (Spain, 36°55′-37°15′N, 2°31-3°40′W). La Caldera is oligotrophic (total phosphorus [TP] < 0.3 µM, [Chl *a*] < 1 µg L^−1^) and receives mineral-nutrient inputs from frequent Saharan dust depositions^62^. The water temperature ranges from 4 °C to 17 °C during the ice-free season and the lake does not stratify^63^. The lake water is highly transparent (*k*_d_ for PAR = 0.05), having a low DOC concentration (<1 mg C L^−1^) that allows >1% UVR to penetrate to the bottom of the lake^54^. Ciliates and heterotrophic nanoflagellates are scarce and were not detected during the present study period.

### Experimental design

An experiment was conducted *in situ* in La Caldera in 2009, from August 27 through September 3. The experiment had a 2 × 2 × 3 factorial design (each treatment in triplicate): two solar radiation treatments [full sunlight (UVR + PAR; 280–700 nm) and only photosynthetic active radiation [PAR; PAR; 400–700 nm)]; two nutrient treatments [ambient nutrient concentration (P-ambient) and P addition (P-added)]; and three temperature treatments [5 °C above ambient temperature (T_+_), ambient temperature (T_=_), and 5 °C below ambient temperature (T_−_)]. The experiment had a split-plot design, with radiation and nutrient treatments at main-plot level and temperature at sub-plot level (Fig. 2S).

Each treatment used three microcosms, i.e. 20-L clear-polythene cylinders. Polyethylene that transmits ~60% [280 nm] and >80% [400–700 nm] was used for the UVR + PAR and PAR treatments, with the addition of a Plexiglas UF3 cover (a long-wave-pass plastic that transmits 90% of PAR but blocks UVR < 390 nm) for the PAR treatments. Each microcosm was filled with water that had been pre-screened through 45-µm nylon mesh for mesozooplankton removal from the photic layer receiving >1% UVR-B. Mesozooplankton was excluded because the volume of the microcosms was not suitable to maintain a grazer community for a week. Hence, we avoided the lack of replicability in the microcosms due to the uneven effect of herbivores on phytoplankton. The microcosms were incubated *in situ* at 0.5 m depth for a week. For the P-added treatments, KH_2_PO_4_ was added to a final concentration of 30 μg P L^−1^, based on previous findings of a maximum response of the microbial food web at this concentration in the high-mountain lake^46^. Microcosms with no added P and no UVR exposure served as controls of nutrient addition and UVR effect. Microcosms were gathered after one week and the contents of each were divided into three samples (250 mL acid-clean glass flasks) for incubation under one of three temperature conditions: *in situ* temperature (T_=_, 17 °C), high temperature (T_+_, 22 °C) and low temperature (T_−_, 12 °C). The incubation of samples under the temperature treatments was performed in the dark for 12 h. After that, the incubations for PP and HBP were performed *in situ*, and the control of radiation quality was achieved using quartz flasks covered by polyethylene plastic (for UVR + PAR treatment) or glass flasks covered by Plexiglas UF3 (for PAR treatment).

### Physical analyses

Vertical profiles of solar radiation in the water column were determined at noon with a BIC compact four-channel underwater radiometer (Biospherical Instruments Inc., CA, USA) (Supplementary Text 1S).

### Biotic and abiotic structural variables

Samples for dissolved inorganic nitrogen (DIN) and TP were analysed on the same day as their collection (See Supplementary Text 2S for laboratory protocol). Up to 500 mL for seston N, or 1 L for seston P (per replicate) were filtered through pre-combusted (1 h at 550 °C) 1.0-μm glass fibre filters (Whatman GF/B) at low pressure (<100 mm Hg). Filters containing sestonic N were dried (24 h at 60 °C) and kept desiccated until N analysis (Supplementary Text 3S). Sestonic P was analysed following the method described for TP. Blanks and standards were performed in all procedures. The sestonic N:P ratio was calculated on a molar basis.

DOC values were determined by filtering the samples through pre-combusted (2 h at 550 °C) glass-fibre filters (Whatman GF⁄F) and acidifying them with HCl (2%). Samples were then measured in a total organic carbon analyser (TOC-V CSH/CSN Shimadzu).

For measurements of the Chl *a* concentration, 0.5–1 L of water from each microcosm were filtered onto Whatman GF/F filters (25 mm in diameter), which were frozen at −20 °C until analysed (See laboratory protocol in Supplementary Text 4S).

PA was determined by the method of Utermöhl^64^ using samples preserved in glass bottles with alkaline Lugol’s solutions (Supplementary Text 5S). No autotrophic picoplankton was found as in previous reports on this ecosystem^40,45^. We did not assess PA after the temperature treatments because the phytoplanktonic generation time exceeded the incubation time (>12 h) at the different temperatures. BA was determined by the 4′, 6-diamidino-2-phenylindole (DAPI) direct-count method described by Porter and Feig^65^ (Supplementary Text 6S).

### Biotic functional variables

HBP was determined by ^3^H-thymidine (specific activity = 52 Ci mmol^−1^, Amersham Pharmacia) incorporation into the bacterial DNA^66^. In brief, ^3^H-thymidine was added to each experimental flask (25 mL) with 24 mL of sample [3 replicates and 1 killed control with neutralized formaldehyde (0.75% w/v final concentration) per treatment] to a final (saturating) concentration of 15.2 nM. Flasks were incubated with the radiotracer *in situ* at 0.5 m for 90 min symmetrically distributed around noon. After incubation, the incorporation of thymidine was stopped by adding neutralized formaldehyde (See laboratory protocol in Supplementary Text 7S).

For PP measurements, sets of four 50-mL flasks (three clear and one dark for each of 12 experimental treatments) added with 0.37 MBq of NaH^14^CO_3_ (specific activity: 310.8 MBq mmol^−1^, DHI, Water and Environment, Germany) were incubated *in situ* at 0.5 m for 5 h symmetrically distributed around noon. All flask sets were kept horizontal throughout the incubations. PP calculations were based on the ^14^C incorporation method^67^ (See laboratory protocol in Supplementary Text 8S).

To assess phytoplankton-bacterioplankton commensalistic relationship, besides PP we measured EOC and the bacterial uptake of freshly produced phytoplankton C exudates (^14^C-Bact). Thus, we used a serial filtration through 1- and 0.2-µm pore-size filters of 25 mm diameter (Nucleopore Whatman) under low pressure (<100 mm Hg) to minimize cell breakage (more details on laboratory procedure in Carrillo *et al*.^68^ and Supplementary Text 8S). We calculated CARB as the quotient between the HBP and the fraction of photosynthetic exudates assimilated by bacterioplankton, following Medina-Sánchez *et al*.^33^ (Supplementary Text 8S):

1 $${\rm{CARB}}\,({\rm{\mu }}{\rm{g}}\,{{\rm{CL}}}^{-1}{{\rm{h}}}^{-1})={\rm{HBP}}\times {({}^{14}{\rm{C}} \mbox{-} {\rm{Bact}}\times {{\rm{EOC}}}^{-1})}^{-1}$$

### Data treatment and statistics

The experimental data (from the functional variables and BA) compiled from the split-plot design were analysed by a repeated-measure ANOVA (MR-ANOVA), which was used to test the radiation × nutrients interactive effect on each response variable as the main-plot effect; the radiation × nutrients × temperature interaction was tested as sub-plot effect using the multivariate tests of Pillai, Hotelling, and Roy, as recommended by Scheiner and Gurevitch^69^. The interaction between radiation and nutrients for the structural variables not subjected to variation as response to the short-term temperature shifts (sestonic N:P, PA and Chl *a*), were tested by two-way ANOVA. Differences between CARB and EOC values under each experimental treatment were determined by t-test.

When a significant interactive effect of the three or two factors, depending on the variable response, was found, *post hoc* tests were applied to determine the effect of each main factor alone^70^. The Fisher LSD test was used to determine significant differences between treatments. Data were checked for normal distribution (with the Kolmorov-Smirnov test), homoscedasticity (Levene’s test), and sphericity (Mauchly’s test, for RM-ANOVA); data were log-transformed when these conditions were not met. Linear regression analyses were used to study the relationship between bacterial variables (BA and HBP) and EOC rates. Statistica 7.0 for Windows (Statsoft 2001) was employed for the statistical analysis.

The effect size of UVR on each functional variable was calculated as:

2 $${\rm{UVR}}\,{\rm{effect}}\,{\rm{size}}=(({\rm{UVR}}-{\rm{PAR}})/{\rm{PAR}})\times 100$$

where UVR and PAR are the mean values in the presence (UVR + PAR) or absence (PAR) of ultraviolet radiation respectively for each nutrient or nutrient and temperature treatment. Propagation errors were used to calculate the variance of UVR-effect size. On the other hand, the effect size of cooling (T_−_) and warming (T_+_) on each functional variable was quantified as:

3 $${\rm{C}}{\rm{o}}{\rm{o}}{\rm{l}}{\rm{i}}{\rm{n}}{\rm{g}}\,{\rm{e}}{\rm{f}}{\rm{f}}{\rm{e}}{\rm{c}}{\rm{t}}\,{\rm{s}}{\rm{i}}{\rm{z}}{\rm{e}}=({{\rm{T}}}_{-}-{{\rm{T}}}_{{\rm{c}}{\rm{o}}{\rm{n}}{\rm{t}}{\rm{r}}{\rm{o}}{\rm{l}}})/{{\rm{T}}}_{{\rm{c}}{\rm{o}}{\rm{n}}{\rm{t}}{\rm{r}}{\rm{o}}{\rm{l}}}\times 100$$

4 $${\rm{Warming}}\,{\rm{effect}}\,{\rm{size}}=({{\rm{T}}}_{+}-{{\rm{T}}}_{{\rm{control}}})/{{\rm{T}}}_{{\rm{control}}}\times 100$$

T_control_ being the samples exposed to ambient temperature (T_=_), and T_−_ or T_+_ being the samples exposed to decrease or increase of 5 °C, respectively. The differences among treatments for UVR effect size or cooling- and warming-effect size were evaluated by a two-way ANOVA.

Direction and strength of the interactive effect of radiation, temperature, and nutrients for each response variable was calculated by comparing the expected additive effect (obtained from the sum of individual effect of each factor) with the non-additive effect (value in the UVR + PAR × P-added × T_+_ or UVR + PAR × P-added × T_−_, for warming or cooling respectively). Piggot *et al*.^6^ was followed to define the nature of interactive effects. The direction and strength of the response of each variable to the interactive effect was calculated as the quotient between the non-additive and expected additive effects. We used propagation errors to calculate the relative magnitude of the interactive effect.

## Supplementary information

Supplementary information.
