# Publisher Correction: Uncoupled phytoplankton-bacterioplankton relationship by multiple drivers interacting at different temporal scales in a high-mountain Mediterranean lake

**DOI:** 10.1038/s41598-020-62863-6

**Published:** 2020-04-10

**Authors:** Cristina Durán-Romero, Juan Manuel Medina-Sánchez, Presentación Carrillo

**Affiliations:** 1 Instituto del Agua, Universidad de Granada, E-18071 Granada, Spain; 2 Departamento de Ecología, Facultad de Ciencias, Universidad de Granada, E-18071 Granada, Spain

Correction to: *Scientific Reports* 10.1038/s41598-019-57269-y, published online 15 January 2020

The original version of this Article contained missing bar chart outlines and missing error bars in all figures.

Additionally, in Figure 1 the figure key was incorrect. The individual groups for “Crysophyceae”, “Bacillariophyceae” and “Dinophyceae” are now in one group “Others”.

As a result, the legend of Figure 1 was incorrect.

“Sestonic N:P ratio (on a molar basis), phytoplankton abundance (PA) and chlorophyll *a* (Chl *a*) concentration under full sunlight (UVR + PAR) and photosynthetically active radiation (PAR) and under ambient phosphorus (P) concentration and P-added conditions. Bars represent the mean values and error bars represent the standard deviation (SD) (n = 3).”

now reads:

“Sestonic N:P ratio (on a molar basis), phytoplankton abundance (PA) and chlorophyll a (Chl a) concentration under full sunlight (UVR + PAR) and photosynthetically active radiation (PAR) and under ambient phosphorus (P) concentration and P-added conditions. In Fig. 1b, Others group includes Cryptophyceae, Bacillariophyceae and Dinophyceae, and represents a small proportion of the total phytoplankton abundance. Bars represent the mean values and error bars represent the standard deviation (SD) (n = 3).”

This has now been corrected in the PDF and HTML versions of the original article.

