# Supplementary material for: Uncoupled phytoplankton-bacterioplankton relationship by multiple drivers interacting at different temporal scales in a high-mountain Mediterranean lake
Source: Sci Rep. 2020 Jan 15;10:350. doi: 10.1038/s41598-019-57269-y (PMC6962384; doi:10.1038/s41598-019-57269-y)
Supplement: Supplementary file 1 — Supplementary information. [file 41598_2019_57269_MOESM1_ESM.pdf]

Supporting information for

Uncoupled phytoplankton-bacterioplankton relationship by multiple drivers interacting at different temporal scales in a high-mountain Mediterranean lake

Cristina Durán-Romero, Juan Manuel Medina-Sánchez, Presentación Carrillo*

*Corresponding author: pcl@ugr.es

Variable	
TN (µmol L^{-1})	22.80 ± 7.60
TP (µmol L^{-1})	0.11 ± 0.05
SRP (µmol L^{-1})	0.06 ± 0.001
DIN (µmol L^{-1})	14.60 ± 7.30
DIN:TP (µmol)	142.10 ± 27.12
DOC (µmol L^{-1})	72.12 ± 16.21
Chl *a* (µg L^{-1})	3.09 ± 0.04
$N:P_{sestonic}$	40.90 ± 7.40
PA (cell mL^{-1})$\times 10^3$	4.88 ± 1.61
PP (µgC L^{-1} h^{-1})	2.02 ± 0.43
BA (cell mL^{-1})$\times 10^5$	5.76 ± 2.13
HBP (µg C L^{-1} h^{-1})	0.067 ± 0.005

Table 1S. Mean values (± standard deviation) of the main chemical and biological variables studied in the water column under the initial conditions of the experiment. TN: total nitrogen; TP: total phosphorus; SRP: soluble reactive phosphorus; DIN: dissolved inorganic nitrogen; DOC: dissolved organic carbon; Chl *a*: chlorophyll *a*; $N:P_{sestonic}$: nitrogen to phosphorus ratio of the seston on a molar basis; PA: phytoplanktonic abundance; PP: primary production; BA: bacterial abundance; HBP: heterotrophic bacterial production.

	N:P$_{sestonic}$		PA		Chl *a*	
	$F_{1,8}$	p	$F_{1,8}$	p	$F_{1,8}$	p
Rad	0.19	**<0.01**	3.73	0.08	0.15	0.70
P	609.3	**<0.001**	93.89	**<0.001**	111.5	**<0.001**
Rad ×P	1.96	0.20	4.92	0.06	1.58	0.24

Table **2S**. Results of the two-way analysis of variance (ANOVA) of the interactive effect of radiation (Rad) and phosphorus (P) addition. Numbers in bold indicate significant interactive effect between the factors. N:P$_{sestonic}$: nitrogen to phosphorus ratio of the seston on a molar basis; PA: phytoplanktonic abundance; Chl *a*: chlorophyll *a* concentration.

	PP		EOC		HBP		BA		CARB	
Main plot effect	$F_{1,8}$	p	$F_{1,8}$	p	$F_{1,8}$	p	$F_{1,8}$	p	$F_{1,8}$	p
Rad	1453	**<0.001**	3669	**<0.001**	1727.85	**<0.001**	34.09	**<0.001**	98.26	**<0.001**
P	9280	**<0.001**	22392	**<0.001**	1.15	0.315	20.39	**<0.01**	439.43	**<0.001**
Rad×P	63.12	**<0.001**	4340	**<0.001**	13.52	**<0.01**	2.77	0.134	66.67	**<0.001**
Sub-plot effect	$F_{2,7}$		$F_{2,7}$		$F_{2,7}$		$F_{2,7}$		$F_{2,7}$	
T	1260	**<0.001**	283.42	**<0.001**	62.54	**<0.001**	15.42	**<0.01**	22.32	**<0.001**
T×Rad	117.6	**<0.001**	31.88	**<0.001**	14.01	**<0.001**	6.65	**<0.05**	5.33	**<0.05**
T×P	175.7	**<0.001**	348.58	**<0.001**	72.42	**<0.001**	4.5	0.055	11.52	**<0.001**
T×Rad×P	357.8	**<0.001**	35.26	**<0.001**	3.47	0.056	7.96	**<0.05**	0.54	0.59

Table 3S. Results of the repeated three-way analysis of variance (RM-ANOVA) of the interactive effect of radiation (Rad), phosphorus (P) addition, and temperature (T). Numbers in bold indicate significant interactive effect among the factors from RM-ANOVA (main-plot effect) or from multivariate tests of Pillai, Hotelling and Roy (sub-plot effect). PP: primary production; EOC: excreted organic carbon; HBP: heterotrophic bacterial production; BA: bacterial abundance; CARB: photosynthetic carbon required by bacterioplankton.

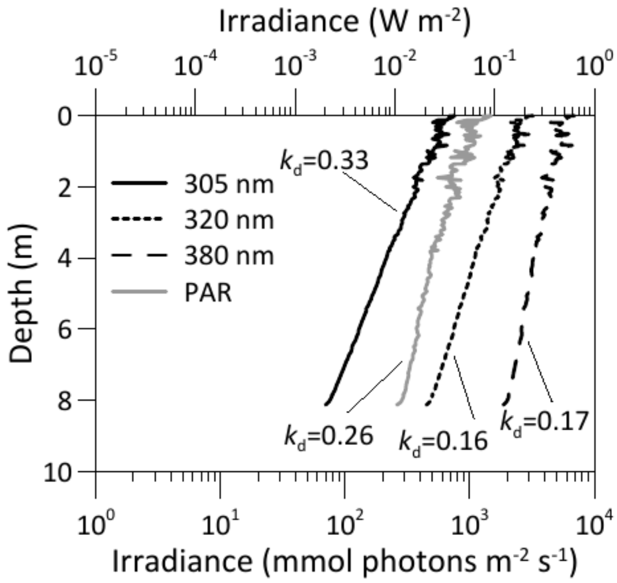

Fig. 1S. Depth profile of the irradiance in the water column at the beginning of the experiment. Irradiance data in the UVR range are expressed in W m^{-2}, PAR is in μmol photons m^{-2} s^{-1}. The diffuse attenuation coefficients (k_d) are also shown.

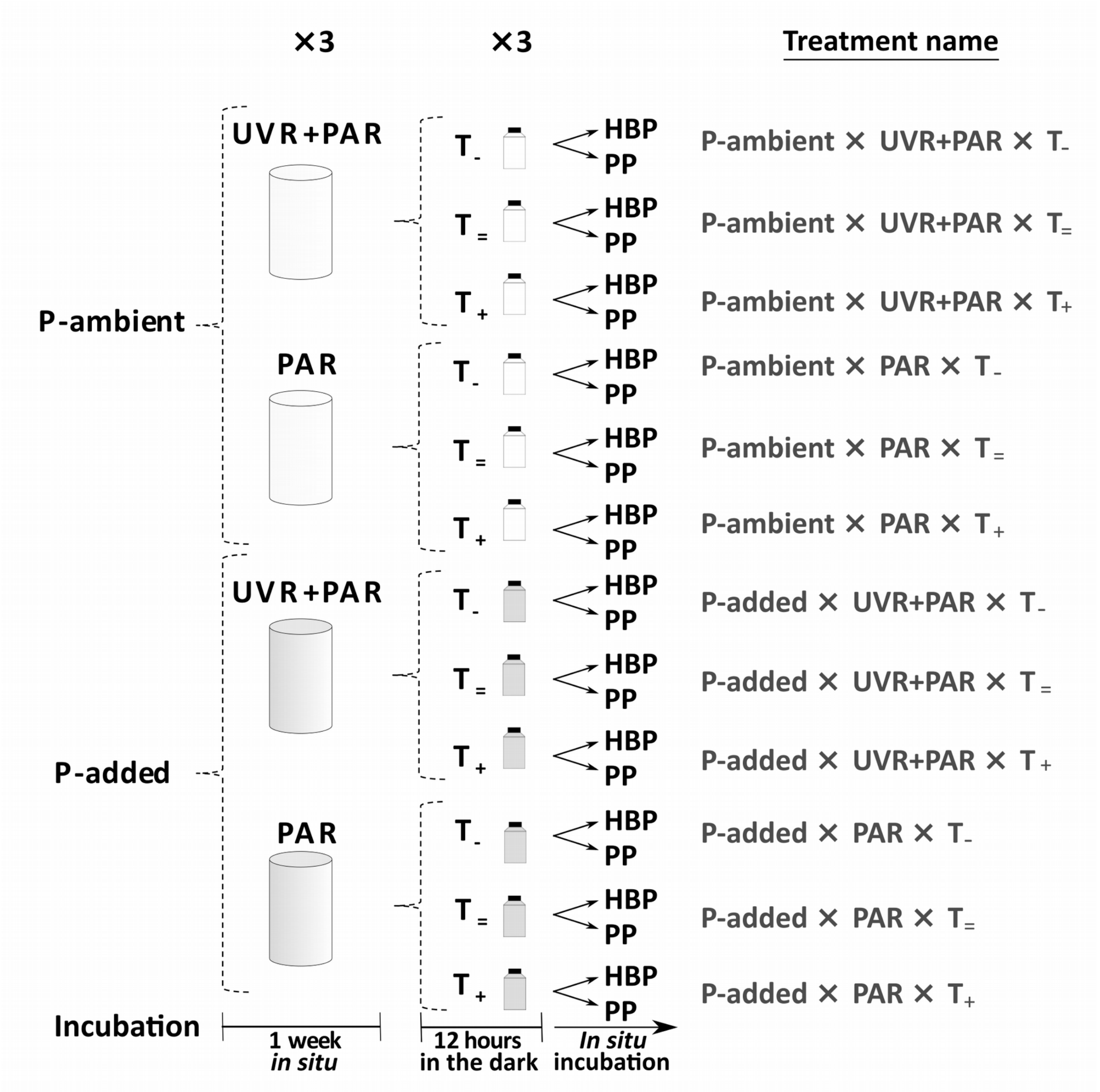

Fig. 2S. Experimental design and experimental treatments nomenclature. UVR+PAR: full sunlight. PAR: photosynthetically active radiation. P-ambient: ambient phosphorus (P) concentration. P-added: increased P concentration. T_-: 5ºC below ambient temperature. $T_=$: ambient temperature. T_+: 5ºC above ambient temperature. HBP: heterotrophic bacterial production. PP: primary production. Samples were incubated (triplicate) *in situ* during 90 min for HBP and 5h for PP.

Supplementary text 1S

A large sample size (pairs irradiance-depth values, n >160) was used, and a good fit (R^2 > 0.95) was found for all regressions.

Supplementary text 2S

DIN was considered the sum of nitrate (NO_3^-), nitrite (NO_2^-), and ammonium (NH_4^+). NO_3^- was analysed by UV spectrophotometric screening, NO_2^- was determined using the sulphanilamide method, and NH_4^+ by the phenol-hypoclorite method[1]. Total nitrogen (TN) and TP were determined by analysing 50-mL aliquots after digestion with a mixture of potassium persulfate, boric acid, and sodium hydroxide at 120ºC for 30 min[1]. NO_3^- and phosphate concentrations in the digested samples were measured following Grasshoff et al.[2].

Supplementary text 3S

A Perkin-Elmer model 2400 CHN elemental analyser (Perkin-Elmer Corporation, Waltham, Massachusetts, USA) was used for samples analysis.

Supplementary text 4S

Samples were thawed and placed in centrifuge tubes (15 mL) with 5 mL of acetone (90%) for 24 h in the dark at 4ºC. Next, the samples were centrifuged, and the fluorescence of the supernatant was measured with a fluorimeter (LS 55 Perkin Elmer, USA). A Chl *a* standard (Chl *a* from spinach, Sigma) was used to transform the fluorescence data into Chl *a* concentrations.

Supplementary text 5S

To quantify PA, 50 mL aliquots were settled in Uthermöhl chambers of 2.6 cm diameter for 48 h to ensure complete sedimentation of the smallest algal species. Cells were counted in

100 randomly selected fields of view at 1000× magnification under an inverted microscope. At least 600 cells of the most abundant algal species were counted in each sample.

Supplementary text 6S

Water samples were fixed with neutralized formaldehyde (2%), stained with DAPI to a final concentration of 2.5 μg mL^{-1}, and then filtered through a 0.2-μm pore-size black polycarbonate Nucleopore Filter. At least 400 cells per sample were counted by epifluorescence microscopy at 1000× magnification (Karl Zeiss AX10).

Supplementary text 7S

Aliquots of 1.5 ml were taken from each replicate and placed in microcentrifuge vials. TCA cold extraction was performed by keeping the vials in ice for 20 min, after which the precipitate was collected by centrifugation (at 16,000*g* for 10 min). Then, vials were rinsed twice with 1.5 mL of 5% TCA to remove any residual unincorporated radioactivity, and scintillation liquid (Ecoscint A) was added for subsequent measurement in an autocalibrated scintillation counter (Beckman LS 6000TA)[3]. The conversion factor 1×10^{18} cell mol^{-1} [4] was used to estimate the number of bacteria produced per mol of incorporated thymidine. The factor 2×10^{-14} g C $cell^{-1}$ [5] was applied to estimate the amount of carbon.

Supplementary text 8S

The ^{14}C measured on the 1μm filters represents the ^{14}C incorporated into phytoplankton cells (PP). Because of the absence of autotrophic picoplankton in this lake, the <1 μm filtrate collected on the 0.2 μm filters represents the ^{14}C incorporated into the heterotrophic bacterioplankton cells (^{14}C-Bact) through the bacterial use of EOC. Thus, the <1 μm filtrate (fraction >0.2 μm + filtrate <0.2 μm) represents EOC. For measurement of the EOC fraction

in filtrate <0.2 μm, we collected aliquots of 4 mL from the filtrate <0.2 μm and they were put into scintillation vials. Filters for PP determination (1μm filters) and ^{14}C-Bact (0.2 μm filters), were put into scintillation vials. Inorganic carbon from scintillation vials was removed by adding 100 μL of 1 N HCl and allowing the vial to remain open in a hood for 24 h. After acidification, a scintillation cocktail (Ecoscint A) was added to all the samples. The amount of carbon was determined from the disintegrations per min (dpm), counted with a scintillation counter equipped with autocalibration (Beckman LS 6000TA). In all calculations, dark values were subtracted from corresponding light values.

CARB may be an upper estimate of the actual bacterial demand for photosynthetic carbon because respired carbon is not included in the ^{14}C-Bact variable and steady state and ^{14}C-isotopic equilibrium with the autochthonous pool is assumed; therefore, the denominator (i.e. ^{14}C-Bact × EOC^{-1}) can be underestimated[6].

References

1. APHA. *Standard methods for the examination of water and wastewater.* (American Public Health Association, 1992).
2. Grasshoff K., Ehrhardt M. & Kremling K. *Methods of seawater analysis*. (Verl. Chem., Weinheim, 1999).
3. Smith D. C. & Azam F. A simple, economical method for measuring bacterial protein synthesis rates in seawater using ^{3}H-leucine. *Mar. Microb. Food Webs* **6**, 107–114 (1992).
4. Bell R. T. Estimating production of heterotrophic bacterioplankton via incorporation of tritiated thymidine. in *Handbook of methods in aquatic microbial ecology* 495–503 (Lewis, 1993).
5. Lee S. & Fuhrman J. A. Relationships between biovolume and biomass of naturally derived marine bacterioplankton. *Deep Sea Res. Part B Oceanogr. Lit. Rev.* **34**, 1069 (1987).
6. Medina-Sánchez, J. M., Villar-Argaiz, M. & Carrillo, P. Solar radiation-nutrient interaction enhances the resource and predation algal control on bacterioplankton: A short-term

experimental study. *Limnol. Oceanogr.* **51**, 913–924 (2006).
